# Poisoning from Corrosive Sublimate Generated in the Mouth from Amalgam Plugs in the Teeth

**Published:** 1873-07

**Authors:** J. Payne

**Affiliations:** Dwight, Ill.


ARTICLE III.—*Poisoning from Corrosive Sublimate generated in the Mouth from Amalgam Plugs in the Teeth.* By J. PAYNE, D.D.S., Dwight, Ill.

Having been invited by an eminent gentleman of the medical profession to attend a convention of the State Medical Society, to submit to its consideration a matter of vital importance to the human family, and being unable to comply with the invitation, I have written this article to lay the matter before the medical profession and ask its co-operation.

The matter which I wished to bring to the notice of the profession is the poisoning of thousands of people all over the world from corrosive sublimate generated in the mouth from amalgam plugs in the teeth. Neither Asiatic cholera, nor small-pox, nor any malarious disease, is doing half the mischief in the world that is being done by this poisoning. Every medical man of any considerable practice has undoubtedly had numerous cases of it, but never knew what it was. The symptoms are so numerous and varied in different cases that it would be impossible to give them all in this short article, but I will say that a person poisoned in this way is liable to be treated for dyspepsia, neuralgia, paralysis, consumption, and numerous throat diseases. The patient gradually wastes away as if going into a decline, and no medicine will afford any relief. In many cases the difficulty steals on so gently as not to excite the least alarm, and continues very gradually for a number of years till the patient becomes a total wreck, while in others the attack comes on violently and the friends and attending physician think the patient is dying, but he will again rally and again be prostrated.

There is such a resemblance in the symptoms to nearly all the diseases to which human flesh is heir, that the physician is led to treat the patient for some disease which seems to be a very clear case, but his patient gets worse. In more than twenty cases that I have had, nearly all had been pronounced by some physician as having consumption. In nearly all the cases there are at times a very bad cough, eyes sunken, and haggard expression and deep blue or dark color under the eyes, invariably a metallic taste in the mouth, water flowing from the mouth in the night while asleep so as to wet the pillow, and in most cases extreme prostration.

I have not time now to detail the manner in which the corrosive sublimate is formed in the mouth, further than to say that the quicksilver in the plugs is driven off by the heat of the mouth in very minute particles, and, combining with the chlorine in the fluids of the mouth, or any saline substance, such as our food, passes into the stomach, and produces slow poisoning. If the State Medical Society will appoint a committee to visit this place, I will show them several cases that will place the matter beyond controversy.

There are some twelve thousand dentists in the United States doing a wholesale business at this poisoning, and I ask the co-operation of the State Medical Society, as guardians of the public health, to assist in getting an act of Congress passed making it a penitentiary offense to place any poisonous substance in teeth that will injure the people.

